# Value of procalcitonin (pct) as diagnostic test of infection in cardiac surgery (cs)

**DOI:** 10.1186/2197-425X-3-S1-A107

**Published:** 2015-10-01

**Authors:** A Pavalascu, T Arce Arias, ML Jimenez Lizarazu, JM Nuñez Martinez, E Tebar Boti

**Affiliations:** Hospital Universitario Vinalopo, Elche, Spain

## Introduction

The systemic inflammatory response syndrome is common after surgery, and it is often difficult to distinguish between infection and inflammation in patients (pts.) undergoing CS. Monitoring PCTs kinetic may be useful to guide antibiotic therapy decisions [1].

## Objectives

To analyse the spontaneous PCTs kinetic after CS and to evaluate the role of PCT in discriminating postoperative infections.

## Methods

A prospective, descriptive study for 4 months (period: Oct 2014 - Jan 2015) in pts. admitted to our ICU. We included adults undergoing emergent, elective or semi-urgent heart valve replacement, coronary artery bypass grafting or aortic aneurysm surgery. Perioperative and outcome data were collected daily for 5 days. The pts. were subdivided in 3 groups: uncomplicated CS group, infectious complications group and non-infectious complications group. Perioperative non-infectious complications were reflected by inotropic and vasoactive drugs used, blood products transfusión, and reintervention for bleeding.

## Results

A total of 84 pts. (76,2% males) were studied. Mean age was 68 ± 10 years. APACHE II on ICU admission was 27 ± 3. The most common CS performed was valve replacement (46,4%). Comorbidities were: diabetes (n = 37), multiple vascular disease (n = 24), and chronic pulmonary disease (n = 20). Mean ICU stay was 3 ± 2 days. Early infections ( < 1 week) occurred in 20,2% pts., the most frequent being respiratory infection (n = 13). Preoperative serum PCT was 0,05 ng/ml (from no detectable [ < 0.05] to 1,58). In pts. with documented infection (n = 17), serum PCT reached peak level at 24-48 hours after surgery, 3,8 ± 1,8 ng/ml (97 %CI; p = 0,001), and decreased progressively after starting antibiotics. Serum PCT levels at 24 and 72 hours after surgery were found to correlate positively with SOFA scores (r = 0.54 and r = 0.66) and APACHE II scores (r = 0.50 and r = 0.55). In non-infectious complications group (n = 51) serum PCT peak level was 1,3 ± 1,02 ng/ml (95 %CI; p < 0,03), registered 24 hours after surgery. Positive correlation with APACHE II scores (r = 0.53 and r = 0.60) at 24 and 72 hours after surgery were found. In uncomplicated CS group (n = 16) serum PCT peak level was 0,83 ± 0,18 ng/ml (90 %CI; p = 0,02), reached at 24 hours after surgery, higher when extracorporeal circulation was used (1,16 ± 0,4 vs. 0,5 ± 0,32), no statistical difference registered.

## Conclusions

Monitoring PCTs kinetic appears to be usefull to guide antibiotic therapy in pts. undergoing CS. During the inmediate postoperative period PCT levels increased, peak level registered at 24 hours. Higher plasma PCT concentration was observed when perioperative non-infectious complications were detected. In the pts. with documented infection, serum PCT continued to increase after surgery until the antibiotic therapy was started.
